#  Biocontrol potential of three novel *Trichoderma* strains: isolation, evaluation and formulation

**DOI:** 10.1007/s13205-013-0150-4

**Published:** 2013-06-30

**Authors:** A. K. Mukherjee, A. Sampath Kumar, S. Kranthi, P. K. Mukherjee

**Affiliations:** 1 Central Institute for Cotton Research, PB 2, Shankar Nagar PO, Nagpur, 440010 Maharashtra India; 2 Present Address: Central Rice Research Institute, Cuttack, Odisha India

**Keywords:** *Trichoderma*, Biological control, Formulation, *Sclerotium delphinii*, Cotton

## Abstract

We have isolated three novel strains of *Trichoderma* (two *T. harzianum* and one *T. atroviride*) from wild mushroom and tree bark, and evaluated their biocontrol potential against *Sclerotium delphinii* infecting cultivated cotton seedlings. *T. harzianum* strain CICR-G, isolated as a natural mycoparasite on a tree-pathogenic *Ganoderma* sp. exhibited the highest disease suppression ability. This isolate was formulated into a talcum-based product and evaluated against the pathogen in non-sterile soil. This isolate conidiated profusely under conditions that are non-conducive for conidiation by three other *Trichoderma* species tested, thus having an added advantage from commercial perspective.

## Introduction

*Trichoderma* spp. are widely used as commercial biofungicides all over the world (Harman 2006; Harman et al. 2004; Howell 2006; Lorito et al. 2010; Schuster and Schmoll 2010; Shoresh et al. 2010; Verma et al. 2007). In India alone, more than 250 commercial formulations are available (Singh et al. 2012), but almost all of them are based on a single strain of *T. viride* (recently reclassified as *T. asperelloides*; Mukherjee et al. 2013b), isolated from rhizosphere (Sankar and Jeyarajan 1996). Soil/rhizosphere has been classically viewed as the main habitat of *Trichoderma*, even though the maximum diversity of this species occurs aboveground e.g., on tree bark and wild mushrooms, and mycotrophy is viewed as the ancestral trait of this genus (Druzhinina et al. 2011). Consequently, only a few strains have been isolated from soil/rhizosphere and used as commercial biopesticides, and the above ground source remained largely unexploited in agriculture, except, perhaps, for a few endophytic strains, such as *T. gamsii* (http://www.clemson.edu/extension/horticulture/fruit_vegetable/peach/diseases/arr_biological.html). In the present study, we have isolated three novel *Trichoderma* strains from wild mushroom and tree bark and evaluated their potential as biocontrol agents. We have also developed a formulation product based on the most effective strain and evaluated this formulation as seed treatment for suppression of seed and root rot of cotton caused by *Sclerotium delphinii*, an emerging pathogen of cultivated cotton (Mukherjee et al. 2013a).

## Results and discussion

Continued commercial success of *Trichoderma* would depend on identification of novel strains adapted to local conditions. Since the diversity of *Trichoderma* is profound on the above-ground, the success of novel strains to be developed as biocontrol products would be greater if the newer isolates are obtained that are naturally mycoparasites, as against collecting a large number of typical saprophytes (from soil) and mass screening. The current study has focused on isolation of *Trichoderma* from wild mushroom and tree bark and evaluation for biocontrol against a newly reported pathogen (*S. delphinii*, MTCC 11568) of cotton.

**Fig. 1:**
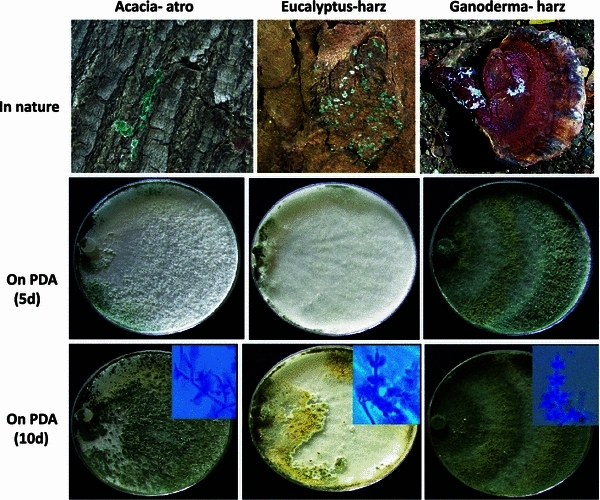
Natural occurrence and cultural characteristics of three isolates of *Trichoderma*. *Top left*: occurrence of *T. atroviride* CICR-A on *Acacia* sp. bark; *Top middle*: occurrence of *T. harzianum* CICR-E on *Eucalyptus* sp. bark; *Top right*: occurrence of *T. harzianum* CICR-G on a basidiocarp of *Ganoderma* sp. *Middle panel*: cultural characteristics on PDA, photographed after 5 days of inoculation. *Lower panel*: cultural characteristics on PDA, photographed after 10 days of inoculation. *Inset*: conidiophore structures observed under microscope

**Fig. 2:**
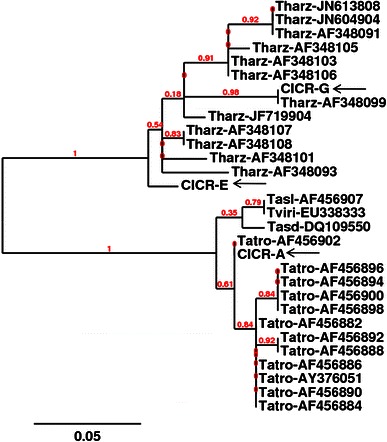
Phylogenetic analysis of *Trichoderma* isolates based on the sequence of the fourth intron of translation elongation factor 1-alpha gene. The positions of new isolates are indicated with *arrows*

Of the three new isolates, *T. harzianum* CICR-G (MTCC 11511) was isolated from a parasitized basidiocarp of a *Ganoderma* sp. that was growing as a parasite on roots of an Acacia tree, *T. harzianum* CICR-E (MTCC 11500) was isolated from the bark of an Eucalyptus tree and *T. atroviride* CICR-A (MTCC 11512) was isolated from the bark of an Acacia tree (Fig. 1). There was large cultural variability among the two *T. harzianum* isolates which were also phylogenetically distantly related (Fig. 2). The Tef1 large (fourth) intron sequence data from all the three isolates have been deposited with GenBank viz. accession nos. KC679853 (*T. harzianum* CICR-G), KC679855 (*T. harzianum* CICR-E) and KC679854 (*T.atroviride* CICR-A).

**Fig. 3:**
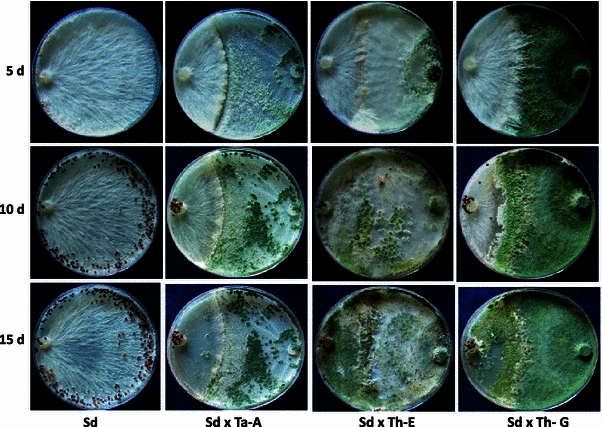
Confrontation assay for antagonism of *Trichoderma* isolates on *Sclerotium delphinii*

**Fig. 4:**
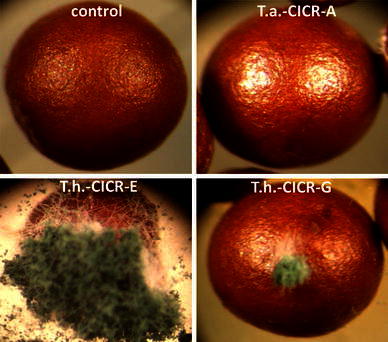
Colonization of sclerotia of *Sclerotium delphinii* by *Trichoderma* isolates, observed under a stereo binocular microscope. Control: a sclerotium from a pure culture

In confrontation assay, both *T. harzianum* CICR-G and *T. harzianum* CICR-E were able to overgrow the test pathogen, but *T. atroviride* CICR-A failed to overgrow *S. delphinii* colony even after prolonged incubation (Fig. 3). Interestingly, the ability to colonize the sclerotia (resting structures of *S. delphinii*) also differed- *T. harzianum* CICR-E being the most effective while *T. atroviride* CICR-A being unable to colonize the sclerotia (Fig. 4).

**Fig. 5:**
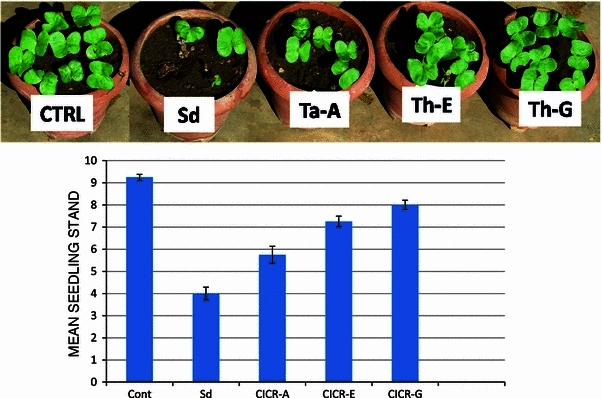
Biological suppression of *Sclerotium delphinii* by *Trichoderma* isolates in cotton. Data are mean of 4 replicates ± SE

The isolates also differed in their ability to suppress *S. delphinii* in sterile soil. *T. harzianum* CICR-G being the most effective, while *T. atroviride* CICR-A being the least effective (Fig. 5). Based on this experiment, *T. harzianum* CICR-G was selected for further studies. It may be noted that even though *T. harzianum* CICE-E was more effective in confrontation assay, *T. harzianum* CICR-G was better as a biocontrol agent in pot soil. This is quite common, as the behaviour of an antagonist in pure culture is many a times different from that in soil where the performance of the bioagent is an outcome of interactions of the antagonist with the pathogen under the influence of several biotic and abiotic factors.

**Fig. 6:**
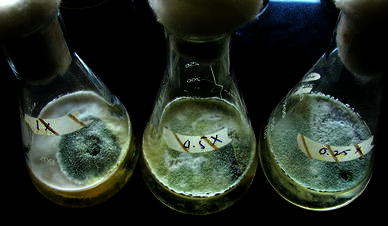
Conidiation of *Trichoderma harzianum* CICR-G on different strengths of PDB after 3 days of incubation. 1×: full strength, 0.5×: half strength, 0.25×: a quarter strength

For developing a formulation product, we assessed the ability of *T. harzianum* CICR-G to conidiate in PDB of varying strengths. Interestingly, this isolate conidiated profusely within 3 days on PDB of one-fourth strength (Fig. 6). After 7 days, the number of conidia produced was 4.5 × 10^10^, 7 × 10^10^ and 4.7 × 10^10^, on 0.25×, 0.5× and 1× PDB, respectively (in a flask with 100 ml medium). We mixed the mat from two flasks (0.5× PDB) per kg talcum powder and after drying and packaging, obtained an initial approximate CFU (colony forming units) count of 10^8^/g formulation product, designated as TrichoCASH 1 % WP.

**Fig. 7:**
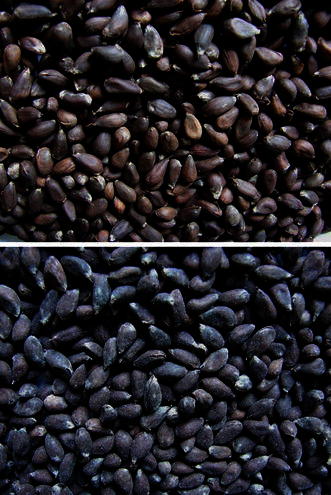
Seed treatment with TrichoCASH 1 % WP. *Top*: untreated cotton seeds; *bottom*: treated seeds

**Fig. 8:**
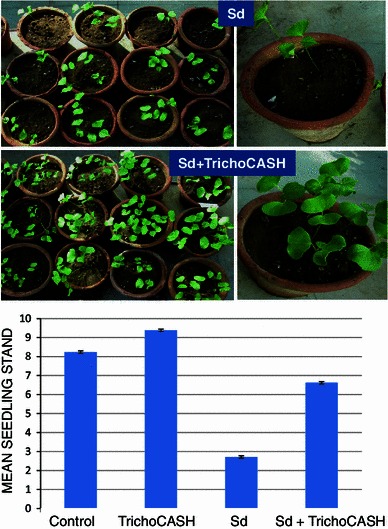
Biological suppression of *Sclerotium delphinii* in cotton in non-sterile soil. Data are mean of 21 replicates ± SE

Wet seed treatment (5 g/kg seeds) provided a uniform coating on acid de-linted cotton seeds (Fig. 7) that were used for sowing in non-sterile soil pre-infested with *S. delphinii*. Treating the seeds with TrichoCASH significantly protected seeds and seedlings from *S. delphinii* infection in non-sterile soil (Fig. 8). The seedling stand in non-sterile soil not pre-infested with *S*. *delphinii* was also significantly higher when seeds were treated with this formulation, as compared to non-treated seeds.

**Fig. 9:**
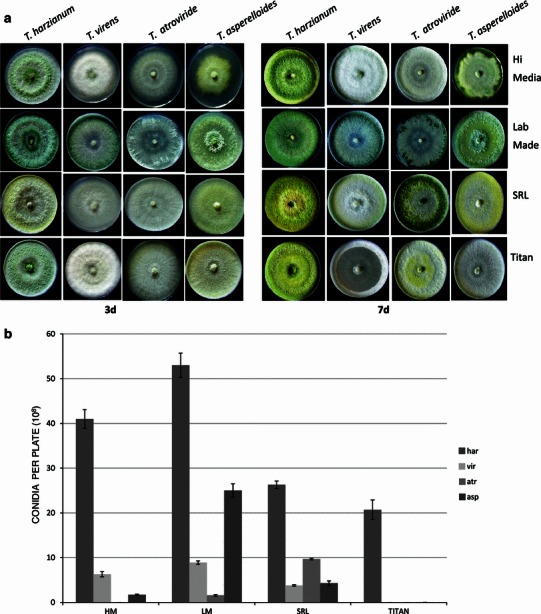
**a** Growth and **b** conidiation of four *Trichoderma* species on PDA from various sources. Data are mean of 3 replicates ± SE

Ability to sporulate under adverse conditions is a desirable trait for biocontrol fungi as this is related to ease of formulation. Of the four different species/strains of *Trichoderma* tested on four different sources of PDA (lab. made, HiMedia, SRL and Titan media), *T. harzianum* CICR-G was least affected by the source of the culture medium (Fig. 9a, b). The laboratory made PDA supported conidiation of all the four species tested, but even on this medium there was wide variation in ability to conidiate; *T.harzianum* CICR-G being the most abundantly sporulating. It is very interesting to note that PDA from different sources have significant effect on conidiation ability of *Trichoderma* spp., and PDA from some commercial sources did not support conidiation of *Trichoderma* (except of *T. harzianum* CICR-G). It needs to be ascertained if the “loss-of-conidiation” of some *Trichoderma* species often observed in laboratories is related to switch to a different source/batch of PDA procured from commercial sources.

*S. delphinii*, like its close relative *S. rolfsii*, is a soil-borne pathogen that over winters through the production of highly melanised sclerotial bodies (Punja 1985; Xu et al. 2008). These attributes make it difficult to control using conventional practices. This pathogen, mostly associated with ornamental crops and certain field crops like groundnut, was hitherto not known to occur in cotton (Edmunds et al. 2003; Farr et al. 2006; Xu et al. 2010). We have recently reported this pathogen to be infecting both seedlings and mature cotton plants in field (Mukherjee et al. 2013a). The soil-borne nature of this pathogen would mean that the pathogen might multiply in soil on crop residues and thus, the incidence would increase with time. In the present study, we have isolated a novel *T. harzianum* strain (naturally occurring as a mycoparasite) that is effective against this emerging pathogen, and also developed a formulation for field applications. The formulation is being tested in multiple locations across India for biocontrol efficacy against seed rot and seedling diseases in cotton.

## Experimental procedure

### Fungal strains and growth conditions

All the three *Trichoderma* strains studied here were isolated from Nagpur, Maharashtra, India, either from infected tree-pathogenic *Ganoderma* sp. or from bark of *Eucalyptus* sp. and *Acacia* sp. The fungi were collected with a sterile cotton swab and the conidia suspended in sterile distilled water. The suspension was plated on potato dextrose agar plates after serial dilution and the isolated colonies were further purified by serial dilution and plating (three times). The pathogen *Sclerotium delphinii* (MTCC 11568) was obtained from our previous studies (Mukherjee et al. 2013a). Routinely, the fungi were grown at ambient temperatures (25–30 °C) on potato dextrose medium prepared in laboratory (200 g potatoes, 20 g dextrose, and 20 g agar–agar, when required, per litre), unless otherwise stated.

### Identification of fungi

The fungal strains were identified based on the sequences of the large sub-unit of translation elongation factor 1-alpha (Tef1) as per standard methods (http://www.isth.info/tools/blast/markers.php). In brief, the large (4th) intron of *tef1* gene was amplified using the primer pair EF1-728F and EF1-986R as recommended, and the product sequenced using an automated DNA sequencer. The species were identified by BLASTN on the NCBI site and the identity confirmed by comparing the sequences with authentic sequences from the GenBank, and a phylogenetic tree constructed on http://www.phylogeny.fr.

### Confrontation assays

Ability of the *Trichoderma* isolates to antagonize the test pathogen *S. delphinii* was assessed using confrontation assay on PDA plates by simultaneous inoculation of both *Trichoderma* and the pathogen near the edge of the plate, placed opposite each other. Ability of *Trichoderma* to overgrow the pathogen colony and also to colonize the sclerotia was recorded.

### Comparative evaluation for biocontrol in green house

*S. delphinii* was grown on autoclaved sorghum grains for 7 days and was inoculated to sterile soil at 2 g per pot containing 2 kg autoclaved black cotton soil. The pots were covered with poly bags for 2 days to facilitate the establishment of the pathogen and after 2 days, 10 seeds of cotton (*Gossypium hirsutum*, variety PKV 081) treated with *Trichoderma* spore suspension (10^7^/ml in 0.5 % aqueous carboxy-methyl cellulose) were sown in each pot. Non-treated seeds sown in pathogen-infested soil served as control. Observation on healthy plant stand was recorded after 10 days.

### Development of a talc-based formulation product

Based on the performance in green house, the best isolate (*T. harzianum* CICR-G) was selected for formulation development and subsequent evaluation. A mycelia disc (6 mm diameter) was inoculated in 100 ml PDB at three concentrations of the medium (1×, 0.5× and 0.25×). Conidiation was counted after 7 days and the mycelial mat along with conidia from 0.5× PDB were mixed thoroughly with autoclaved talcum powder pre-treated with 0.5 % CMC (5 g CMC dissolved in 100 ml water mixed with 1 kg talcum powder). The mix was air-dried in a laminar flow hood and the colony forming units were counted on PDA amended with 100 mg/L rose Bengal, after serial dilution. The formulation was named as TrichoCASH 1 % WP.

### Evaluation of TrichoCASH in green house

Five g of TrichoCASH was taken in a poly bag, and 25 ml water was added to make a slurry. Cotton seeds were treated with this slurry (@5 g/kg seeds) and seeds dried in shade before sowing (10 seeds per pot) in non-autoclaved pot soil (5 kg capacity pots) pre-infested with *S. delphinii* as described above. Observations on healthy seedlings were taken after 15 days.

### Effect of source of medium on conidiation

We have earlier observed that certain strains of *Trichoderma* did not conidiate in PDA from various commercial sources, thus posing a limitation in commercial formulations (Mukherjee PK, unpublished). Hence, we evaluated ability of this strain to conidiate on PDA from different commercial sources, vis-à-vis some other commonly used *Trichoderma* species (*T. atroviride*- this study, *T. asperelloides*, *T. virens*- both kindly gifted by Dr. Ashis Das, NRCC, Nagpur). Mycelial discs were inoculated in the centre of culture plates containing PDA from different commercial sources (Table 1) and observations on conidia production was recorded after 7 days incubation at ambient temperatures. Spores were counted using a hemocytometer, after appropriate dilution.

**Table 1:** Media/components used in this study

Media/component	Manufacturer	Cat. no.	Batch no.	Date of Mfg	Expiry date
PDA (infusion from 200 g boiled potatoes), 20 g dextrose (Hi Media), 20 g agar–agar (Hi Media) in 1 litre RO water pH 6.5	In-house	NA	NA	NA	NA
PDA 39 g/L RO water pH 6.5	Hi Media Laboratories, Mumbai	MU096	0000138972	March 2012	March 2015
PDA 39 g/L RO water pH 6.5	SRL Laboratories, Mumbai	PM015	10062523	June 2012	March 2015
PDA 39 g/L RO water pH 6.5	Titan Biotech, Bhiwandi, Rajasthan	TMV344	V3411112	Not mentioned	November 2014
Dextrose	Hi Media Laboratories, Mumbai	RM077	0000044659	March 2009	Not mentioned
Agar–Agar type I	Hi Media Laboratories, Mumbai	RM666	0000053188	February 2009	February 2014

## References

[CR1] Druzhinina IS, Seidl-Seiboth V, Herrera-Estrella A, Horwitz BA, Kenerley CM, Monte E, Mukherjee PK, Zeilinger S, Grigoriev IV, Kubicek CP (2011). *Trichoderma*: the genomics of opportunistic success. Nat Rev Microbiol.

[CR2] Edmunds BA, Gleason ML, Wegulo SN (2003). Resistance of hosta cultivars to petiole rot caused by *Sclerotium rolfsii* var. *delphinii*. Hort Technol.

[CR7] Farr DF, Rossman AY, Palm ME, McCray, EB (2006) Fungal Databases. Systematic Botany & Mycology Laboratory, ARS, USDA. http://nt.ars-grin.gov/fungaldatabases/

[CR3] Harman GE (2006). Overview of mechanisms and uses of *Trichoderma* spp.. Phytopathology.

[CR4] Harman GE, Howell CR, Viterbo A, Chet I, Lorito M (2004). *Trichoderma* species: opportunistic, avirulent plant symbionts. Nat Rev Microbiol.

[CR5] Howell CR (2006). Understanding the mechanisms employed by *Trichoderma**virens* to effect biological control of cotton diseases. Phytopathology.

[CR6] Lorito M, Woo SL, Harman GE, Monte E (2010). Translational research on *Trichoderma*: from omics to the field. Annu Rev Phytopathol.

[CR8] Mukherjee AK, Mukherjee PK, Kranthi S (2013a) *Sclerotium delphinii* infecting cultivated cotton in India- a first record. New Disease Reports (Submitted)

[CR9] Mukherjee PK, Mukherjee AK, Kranthi S (2013b) Reclassification of *Trichoderma viride* (TNAU), the most widely used commercial biofungicide in India, as *Trichoderma asperelloides*. Open Biotechnol J 7:7–9. doi:10.2174/1874070701307010007

[CR10] Punja ZK (1985). The biology, ecology, and control of *Sclerotium rolfsii*. Annu Rev Phytopathol.

[CR11] Sankar P, Jeyarajan R (1996). Seed treatment formulation of *Trichoderma* and *Gliocladium* for biological control of *Macrophomina phaseolina* in sesamum. Indian Phytopath.

[CR12] Schuster A, Schmoll M (2010). Biology and biotechnology of *Trichoderma*. Appl Microbiol Biotechnol.

[CR13] Shoresh M, Harman GE, Mastouri F (2010). Induced systemic resistance and plant responses to fungal biocontrol agents. Annu Rev Phytopathol.

[CR14] Singh HB, Singh BN, Singh SP and Sarma BK (2012) Exploring different avenues of *Trichoderma* as a potent bio-fungicidal and plant growth promoting candidate- an overview. Rev Plant Pathol 5:315–426, Scientific Publishers (India), Jodhpur

[CR15] Verma M, Brar S, Tyagi R, Surampalli R, Valero J (2007). Antagonistic fungi, *Trichoderma* spp.: panoply of biological control. Biochem Eng J.

[CR16] Xu Z, Gleason ML, Mueller DS, Esker PD, Bradley CA, Buck JW, Benson DM, Dixon PM, Monteiro JEBA (2008). Overwintering of *Sclerotium rolfsii* and *S. rolfsii* var. *delphinii* in different latitudes of the United States. Plant Dis.

[CR17] Xu Z, Harrington TC, Gleason ML, Batzer JC (2010). Phylogenetic placement of plant pathogenic *Sclerotium* species among teleomorph genera. Mycologia.

